# Use of serial smartphone-based assessments to characterize diverse neuropsychiatric symptom trajectories in a large trauma survivor cohort

**DOI:** 10.1038/s41398-022-02289-y

**Published:** 2023-01-07

**Authors:** Francesca L. Beaudoin, Xinming An, Archana Basu, Yinyao Ji, Mochuan Liu, Ronald C. Kessler, Robert F. Doughtery, Donglin Zeng, Kenneth A. Bollen, Stacey L. House, Jennifer S. Stevens, Thomas C. Neylan, Gari D. Clifford, Tanja Jovanovic, Sarah D. Linnstaedt, Laura T. Germine, Scott L. Rauch, John P. Haran, Alan B. Storrow, Christopher Lewandowski, Paul I. Musey, Phyllis L. Hendry, Sophia Sheikh, Christopher W. Jones, Brittany E. Punches, Michael C. Kurz, Robert A. Swor, Vishnu P. Murty, Meghan E. McGrath, Lauren A. Hudak, Jose L. Pascual, Elizabeth M. Datner, Anna M. Chang, Claire Pearson, David A. Peak, Roland C. Merchant, Robert M. Domeier, Niels K. Rathlev, Brian J. O’ Neil, Paulina Sergot, Leon D. Sanchez, Steven E. Bruce, Justin T. Baker, Jutta Joormann, Mark W. Miller, Robert H. Pietrzak, Deanna M. Barch, Diego A. Pizzagalli, John F. Sheridan, Jordan W. Smoller, Steven E. Harte, James M. Elliott, Karestan C. Koenen, Kerry J. Ressler, Samuel A. McLean

**Affiliations:** 1 Department of Epidemiology, Brown University, Providence, RI USA; 2 Department of Emergency Medicine, Brown University, Providence, RI USA; 3 Institute for Trauma Recovery, Department of Anesthesiology, University of North Carolina at Chapel Hill, Chapel Hill, NC USA; 4 Department of Epidemiology, Harvard T.H. Chan School of Public Health, Harvard University, Boston, MA USA; 5 Institute for Trauma Recovery, Department of Psychiatry, University of North Carolina at Chapel Hill, Chapel Hill, NC USA; 6 Department of Biostatistics, Gillings School of Global Public Health, University of North Carolina, Chapel Hill, NC USA; 7 Department of Health Care Policy, Harvard Medical School, Boston, MA USA; 8 Mindstrong Health, Mountain View, CA USA; 9 Department of Psychology and Neuroscience & Department of Sociology, University of North Carolina at Chapel Hill, Chapel Hill, NC USA; 10 Department of Emergency Medicine, Washington University School of Medicine, St. Louis, MO USA; 11 Department of Psychiatry and Behavioral Sciences, Emory University School of Medicine, Atlanta, GA USA; 12 Departments of Psychiatry and Neurology, University of California San Francisco, San Francisco, CA USA; 13 Department of Biomedical Informatics, Emory University School of Medicine, Atlanta, GA USA; 14 Department of Biomedical Engineering, Georgia Institute of Technology and Emory University, Atlanta, GA USA; 15 Department of Psychiatry and Behavioral Neurosciences, Wayne State University, Detroit, MA USA; 16 Institute for Technology in Psychiatry, McLean Hospital, Belmont, MA USA; 17 The Many Brains Project, Belmont, MA USA; 18 Department of Psychiatry, Harvard Medical School, Boston, MA USA; 19 Department of Psychiatry, McLean Hospital, Belmont, MA USA; 20 Department of Emergency Medicine, University of Massachusetts Medical School, Worcester, MA USA; 21 Department of Emergency Medicine, Vanderbilt University Medical Center, Nashville, TN USA; 22 Department of Emergency Medicine, Henry Ford Health System, Detroit, MI USA; 23 Department of Emergency Medicine, Indiana University School of Medicine, Indianapolis, IN USA; 24 Department of Emergency Medicine, University of Florida College of Medicine -Jacksonville, Jacksonville, FL USA; 25 Department of Emergency Medicine, Cooper Medical School of Rowan University, Camden, NJ USA; 26 Department of Emergency Medicine, University of Cincinnati College of Medicine, Cincinnati, OH USA; 27 College of Nursing, University of Cincinnati, Cincinnati, OH USA; 28 Department of Emergency Medicine, University of Alabama School of Medicine, Birmingham, AL USA; 29 Department of Surgery, Division of Acute Care Surgery, University of Alabama School of Medicine, Birmingham, AL USA; 30 Center for Injury Science, University of Alabama at Birmingham, Birmingham, AL USA; 31 Department of Emergency Medicine, Oakland University William Beaumont School of Medicine, Rochester, MI USA; 32 Department of Psychology, Temple University, Philadelphia, PA USA; 33 Department of Emergency Medicine, Boston Medical Center, Boston, MA USA; 34 Department of Emergency Medicine, Emory University School of Medicine, Atlanta, GA USA; 35 Department of Surgery, Department of Neurosurgery, University of Pennsylvania, Philadelphia, PA USA; 36 Perelman School of Medicine, University of Pennsylvania, Philadelphia, PA USA; 37 Department of Emergency Medicine, Einstein Healthcare Network, Philadelphia, PA USA; 38 Department of Emergency Medicine, Sidney Kimmel Medical College, Thomas Jefferson University, Philadelphia, PA USA; 39 Department of Emergency Medicine, Jefferson University Hospitals, Philadelphia, PA USA; 40 Department of Emergency Medicine, Wayne State University, Detroit, MI USA; 41 Department of Emergency Medicine, Massachusetts General Hospital, Boston, MA USA; 42 Department of Emergency Medicine, Brigham and Women’s Hospital, Boston, MA USA; 43 Department of Emergency Medicine, Saint Joseph Mercy Hospital, Ypsilanti, MI USA; 44 Department of Emergency Medicine, University of Massachusetts Medical School-Baystate, Springfield, MA USA; 45 Department of Emergency Medicine, McGovern Medical School, University of Texas Health, Houston, TX USA; 46 Department of Emergency Medicine, Beth Israel Deaconess Medical Center, Boston, MA USA; 47 Department of Emergency Medicine, Harvard Medical School, Boston, MA USA; 48 Department of Psychological Sciences, University of Missouri - St. Louis, St. Louis, MO USA; 49 Department of Psychology, Yale University, West Haven, CT USA; 50 National Center for PTSD, Behavioral Science Division, VA Boston Healthcare System, Boston, MA USA; 51 Department of Psychiatry, Boston University School of Medicine, Boston, MA USA; 52 National Center for PTSD, Clinical Neurosciences Division, VA Connecticut Healthcare System, West Haven, CT USA; 53 Department of Psychiatry, Yale School of Medicine, West Haven, CT USA; 54 Department of Psychological & Brain Sciences, Washington University in St. Louis, St. Louis, MO USA; 55 Division of Depression and Anxiety, McLean Hospital, Belmont, MA USA; 56 Department of Biosciences, OSU Wexner Medical Center, Columbus, OH USA; 57 Institute for Behavioral Medicine Research, OSU Wexner Medical Center, Columbus, OH USA; 58 Department of Psychiatry, Psychiatric and Neurodevelopmental Genetics Unit, Massachusetts General Hospital, Boston, MA USA; 59 Stanley Center for Psychiatric Research, Broad Institute, Cambridge, MA USA; 60 Department of Anesthesiology, University of Michigan Medical School, Ann Arbor, MI USA; 61 Department of Internal Medicine-Rheumatology, University of Michigan Medical School, Ann Arbor, MI USA; 62 Kolling Institute of Medical Research, University of Sydney, St Leonards, NSW Australia; 63 Faculty of Medicine and Health, University of Sydney, Northern Sydney Local, Health District, NSW Australia; 64 Physical Therapy & Human Movement Sciences, Feinberg School of Medicine, Northwestern University, Chicago, IL USA; 65 Department of Emergency Medicine, University of North Carolina at Chapel Hill, Chapel Hill, NC USA

**Keywords:** Pathogenesis, Depression

## Abstract

The authors sought to characterize adverse posttraumatic neuropsychiatric sequelae (APNS) symptom trajectories across ten symptom domains (pain, depression, sleep, nightmares, avoidance, re-experiencing, anxiety, hyperarousal, somatic, and mental/fatigue symptoms) in a large, diverse, understudied sample of motor vehicle collision (MVC) survivors. More than two thousand MVC survivors were enrolled in the emergency department (ED) and completed a rotating battery of brief smartphone-based surveys over a 2-month period. Measurement models developed from survey item responses were used in latent growth curve/mixture modeling to characterize homogeneous symptom trajectories. Associations between individual trajectories and pre-trauma and peritraumatic characteristics and traditional outcomes were compared, along with associations within and between trajectories. APNS across all ten symptom domains were common in the first two months after trauma. Many risk factors and associations with high symptom burden trajectories were shared across domains. Both across and within traditional diagnostic boundaries, APNS trajectory intercepts, and slopes were substantially correlated. Across all domains, symptom severity in the immediate aftermath of trauma (trajectory intercepts) had the greatest influence on the outcome. An interactive data visualization tool was developed to allow readers to explore relationships of interest between individual characteristics, symptom trajectories, and traditional outcomes (http://itr.med.unc.edu/aurora/parcoord/). Individuals presenting to the ED after MVC commonly experience a broad constellation of adverse posttraumatic symptoms. Many risk factors for diverse APNS are shared. Individuals diagnosed with a single traditional outcome should be screened for others. The utility of multidimensional categorizations that characterize individuals across traditional diagnostic domains should be explored.

## Introduction

One of the most common life-threatening trauma exposures in developed countries are motor vehicle collisions (MVCs) [1, 2]. Although >90% of individuals who present to the emergency department (ED) for care after an MVC are discharged to home after evaluation [3], adverse posttraumatic neuropsychiatric sequelae (APNS) are common in this population and result in substantial morbidity [4–13].

The definitions, boundaries, and even putative pathogenesis of traditionally defined APNS developed within the spheres of particular medical subspecialties (e.g., posttraumatic stress disorder—psychiatry, “post-concussion syndrome”—neurosurgery/neurology, chronic pain —anesthesiology) [14]. Similarly, the great majority of research studies of APNS have assessed outcomes from the lens of specific clinical domains, typically focusing on a single traditional outcome. However, increasing evidence suggests that trauma survivors experience patterns of co-occurring symptoms across a number of traditional diagnostic categories and that symptoms across classifications may share overlapping pathogenesis and/or biology [11, 14, 15]. If this is the case, then limiting posttraumatic outcome assessments to one or two traditional diagnoses may poorly represent the trauma survivor experience. Such categorization may also discard phenotypic information which could inform prognosis and treatment.

In this analysis, we examined the post-traumatic trajectories of ten common symptom domains in more than two thousand individuals who presented to the ED after an MVC [16]. These transdiagnostic symptom domains span more traditional diagnostic categories, including posttraumatic stress disorder, depression, pain, and post-concussion syndrome. Repeated symptom assessments were performed using a rotating battery of brief smartphone-based surveys during the initial eight weeks after trauma, a key period of symptom persistence vs. recovery [14]. Measurement models developed from survey item responses were used in latent growth curve/mixture modeling to first establish homogeneous trajectory classes for each symptom over time. We then aimed to explore associations between each symptom trajectory and: [1] participant characteristics (pre-MVC and peri-traumatic) [2], traditional diagnostic outcomes, and [3] other symptom trajectories (e.g., sleep vs. hyperarousal symptoms). In addition, a web-based interactive visualization tool was developed to allow researchers and stakeholders to explore relationships between pre-trauma, trauma-related, and post-traumatic characteristics and symptom trajectories/traditional outcomes of interest (http://itr.med.unc.edu/aurora/parcoord/). We hypothesized that a broad array of post-traumatic symptoms would be common and that moderate to strong correlations would exist between symptom trajectories and traditional diagnostic categories.

## Methods

### Study overview and eligibility criteria

The National Institute of Mental Health initiated a collaborative study, the AURORA (Advancing Understanding of RecOvery afteR traumA) study, to collect a combination of prospective genomic, neuroimaging, psychophysical, physiological, neurocognitive, digital phenotype, and self-report data from an enriched sample of of trauma survivors recruited from EDs in the early aftermath of trauma. The full methodology of the AURORA study has been published elsewhere [14]. In this report, we analyzed a sub-cohort of AURORA participants whose traumatic exposure was an MVC. AURORA enrollment began in September 2017, participants involved in an MVC who had completed the 8-week assessment by the beginning of January 2019 were included in this analysis. Individuals were eligible to participate if they presented to one of 27 EDs within 72 h of the MVC, were aged 18–65, were able to speak and read English, were able to follow the study protocol, and planned to have a smartphone for the next year. Individuals were excluded if they had a solid organ injury Grade >1 per the American Association for the Surgery of Trauma, significant hemorrhage, operative intervention, or were likely to be admitted for >72 h. A total of 3039 patients met all these criteria, provided informed consent, and completed our baseline assessment while still in the ED. Of the 3039 patients enrolled at baseline, 2097 completed the 8-week assessment post-ED enrollment and were included in this analysis. Compared to those who withdrew or were lost to follow-up from the study, participants who continued in the study were older and more likely to be non-Hispanic White. No differences were observed between pre-trauma pain, depression, or somatic symptoms (Supplemental Table 6).

### Outcome measures

Sociodemographic, pre-trauma (i.e., before the MVC leading to the ED evaluation), and peri-traumatic characteristics (i.e., symptoms assessed in the ED) were assessed via survey items [14]. Following the baseline visit in the ED, participants responded to a rotating battery of smartphone-based “flash” questionnaires to assess ten APNS constructs. The ten APNS constructs were selected based on a review of extant literature and expert consensus: [14] pain [17, 18], depressive symptoms [19–22], sleep discontinuity [23], nightmares [24–26], somatic symptoms [17, 27], concentration/thinking/fatigue [28–31], avoidance, re-experiencing anxiety [32, 33], and hyperarousal [34–37]. Each survey item was administered at six timepoints within the first 8 weeks post-trauma (i.e., after the MVC) using the Mindstrong Discovery™ application. For each APNS construct, the corresponding survey items, their response options, and the study day on which they were administered are shown in Supplementary Table 1. Participants also completed assessments of traditional APNS outcomes at 8 weeks following the traumatic event. These included: pain, post-traumatic stress symptoms, post-traumatic somatic symptoms (often referred to as “post-concussive symptoms”), anxiety, and depression. The pain was assessed using the numerical rating scale (NRS), with moderate to severe pain defined as an NRS > 3; somatic symptoms were assessed with the Rivermead questionnaire; post-traumatic stress symptoms were assessed using the Impact of Event Scale—Revised, Anxiety was assessed with the PROMIS Anxiety Bank, and Depression was assessed using the PROMIS Depression Short Form 8b.

### Data analysis

Our overall goal was to quantitatively and then qualitatively describe different trajectories (symptoms over time) across each of the APNS domains for our study cohort using latent growth curve modeling. First, we developed joint measurement models that included all six timepoints, for each of the ten latent constructs. The temporal correlations of a given indicator variable were generally not fully explained by such joint models, therefore temporal correlations of error terms were introduced to account for autocorrelations. Measurement model fit was evaluated via mean square error of approximation, comparative fit index, standardized root mean square residual, and Tucker–Lewis index (Supplementary Table 2). Measurement models with good fit were developed for each timepoint and nested likelihood tests were performed to confirm measurement model structure invariance (Supplementary Table 2). Strong (or scalar) invariance across time was assessed to ensure that factor loading structures and intercept (or mean) parameters underlying indicator variables were the same and factor loading parameters were equivalent. Measurement invariance was tested using the likelihood ratio test. It was expected that some of the model parameters would not satisfy measurement invariance especially when the sample size is large. If the differences were less than 5% and could be explained by substantive reasons, a measurement model with partial invariance was used for the rest of the analyses. One (partial) strong invariance was established, and the growth trajectory pattern of each latent construct was modeled using the embedded measurement models as time-specific factors (Supplementary Table 3). Different trajectory patterns across time, such as linear, quadratic, and piecewise, were explored for each latent construct, and log-likelihood ratio test or comparative model fit indices were used to select the best functional form. The result of these analyses was, for each participant and each latent construct, an intercept and one or two slopes describing that individual’s trajectory for that outcome during the eight weeks after trauma. More than one slope was necessary when the functional form of the trajectory (e.g., linear vs. piecewise) varied over time, such that slopes were modeled piecewise with a knot at the third time point around three weeks.

After the best functional form of each latent growth curve model was identified for each construct, growth mixture modeling was used to estimate latent trajectory classes. The number of latent classes ranged from 1 to 4 and was determined by model fit, the Bayesian information criterion (BIC), the percentage of participants in each latent class, and clinical relevance. Models with convergence issues or with latent classes with less than 5% participants were excluded first. Next, the BIC was used to select the best model. For models with similar BICs, clinical relevance was used to guide the final model selection (Supplementary Table 8). In order to successfully estimate these models a variety of modeling strategies were employed including constraining variance and covariance parameters to be the same across all latent classes, setting small negative variances that were non-significant to zero, and simplifying the model by removing the error correlation structure.

For each construct, participants were assigned probabilities of class inclusion in each latent class, and a single class assignment was made based on the participant’s highest probability class for that outcome. For each participant and each outcome, these results provided a class assignment for their highest probability trajectory category. We assigned qualitative descriptors to each of the trajectories based on visual inspection of the data (e.g, low symptom trajectory and high symptoms followed by recovery). Associations between demographic and pre-trauma characteristics and class memberships were then evaluated, as well as correlations between intercepts and slopes across domains. A web-based interactive visualization tool was also developed to allow investigators to explore relationships between individual trajectories, traditional outcomes, and pre-trauma and trauma-related characteristics http://itr.med.unc.edu/aurora/parcoord/ (see supplemental: Interactive tool for details). Data were treated as missing at random and analyses confirmed that missingness was not related to the APNS constructs (see supplement missing data for details).

## Results

### Participants

A majority of participants were female, Non-Hispanic Black, had completed at least some college, had an income ≤ $35,000/year, were employed (Table 1), were younger (average age < 35), and had minor (i.e., abbreviated injury scale score of (1) injuries (e.g., musculoskeletal strain). Participants were asked to recall pain, somatic symptoms, depression, and anxiety symptoms in the week before the trauma. In the month before the trauma, approximately 32% of participants reported experiencing at least some pain, 3.5% reported any somatic symptoms, 10.7% reported depressive symptoms, and 5% reported anxiety symptoms. The vast majority (>95%) of participants were discharged home after ED evaluation.

**Table 1:** Characteristics of the overall sample and of individuals with specific symptom trajectories.

		Pain					Depression				Reexperience				
	Overall (*n* = 2097)	High (*n* = 464)	High with ↓ (*n* = 777)	High with ↓ ↓ (*n* = 452)	Low (*n* = 181)	*P*-value	High with ↑ (*n* = 208)	Moderate (*n* = 629)	Low ↓ (*n* = 928)	*P*-value	High (*n* = 193)	High with ↓↓ (*n* = 102)	High with ↓ (*n* = 832)	Low (*n* = 680)	*P*-value
Age	34.60 (12.7)	37.70 (13.0)	33.84 (12.4)	31.88 (11.5)	33.72 (13.0)	<0.001	33.61 (12.1)	33.80 (12.2)	35.55 (13.1)	0.032	35.07 (12.2)	37.06 (12.7)	35.11 (12.8)	33.36 (12.5)	0.002
Sex at Birth (Male)	760 (36.2%)	127 (27.4%)	291 (37.5%)	152 (33.6%)	89 (49.2%)	<0.001	60 (28.8%)	209 (33.2%)	343 (37.0%)	0.054	54 (28.0%)	17 (16.7%)	253 (30.4%)	309 (45.4%)	<0.001
Race/Ethnicity						<0.001				0.054					<0.001
Hispanic	257 (12.3%)	54 (11.6%)	101 (13.0%)	56 (12.4%)	18 (9.9%)		27 (13.0%)	95 (15.1%)	99 (10.7%)		24 (12.4%)	8 (7.8%)	120 (14.4%)	74 (10.9%)	
Non-Hispanic White	647 (30.9%)	110 (23.7%)	219 (28.2%)	171 (37.8%)	80 (44.2%)		61 (29.3%)	201 (32.0%)	287 (30.9%)		45 (23.3%)	39 (38.2%)	227 (27.3%)	250 (36.8%)	
Non-Hispanic Black	1102 (52.6%)	285 (61.4%)	421 (54.2%)	203 (44.9%)	71 (39.2%)		112 (53.8%)	307 (48.8%)	501 (54.0%)		115 (59.6%)	51 (50.0%)	451 (54.2%)	325 (47.8%)	
Non-Hispanic Other	81 (3.9%)	11 (2.4%)	33 (4.2%)	20 (4.4%)	11 (6.1%)		5 (2.4%)	24 (3.8%)	37 (4.0%)		7 (3.6%)	3 (2.9%)	29 (3.5%)	30 (4.4%)	
Education (High School or Less)	689 (32.9%)	163 (35.1%)	276 (35.5%)	128 (28.3%)	49 (27.1%)	0.014	82 (39.4%)	218 (34.7%)	274 (29.5%)	0.008	81 (42.0%)	18 (17.6%)	298 (35.8%)	195 (28.7%)	<0.001
Income (<=$35,000)	981 (46.8%)	259 (55.8%)	381 (49.0%)	220 (48.7%)	70 (38.7%)	<0.001	125 (60.1%)	328 (52.1%)	450 (48.5%)	<0.001	114 (59.1%)	52 (51.0%)	444 (53.4%)	304 (44.7%)	<0.001
Pre-Trauma Pain	662 (31.6%)	232 (50.0%)	250 (32.2%)	92 (20.4%)	21 (11.6%)	<0.001	104 (50.0%)	233 (37.0%)	229 (24.7%)	<0.001	94 (48.7%)	33 (32.4%)	314 (37.7%)	142 (20.9%)	<0.001
Pre-Trauma somatic symptoms^a^	3.90 (3.5)	4.91 (3.9)	4.10 (3.6)	3.34 (3.2)	2.67 (2.7)	<0.001	5.59 (3.9)	5.05 (3.7)	2.93 (3.0)	<0.001	5.12 (4.2)	4.14 (3.6)	4.60 (3.7)	2.88 (2.9)	<0.001
Pre-Trauma Depression	48.31 (10.7)	50.22 (11.9)	48.56 (10.4)	47.13 (10.1)	46.73 (8.8)	<0.001	56.64 (13.1)	51.68 (10.7)	44.51 (8.2)	<0.001	52.95 (13.4)	48.92 (9.4)	49.88 (10.9)	45.58 (9.0)	<0.001
Pre-Trauma Anxiety symptoms	5.02 (4.7)	6.15 (5.1)	5.03 (4.5)	4.55 (4.5)	4.05 (3.8)	<0.001	8.32 (5.3)	6.07 (4.7)	3.69 (3.9)	<0.001	7.12 (5.3)	5.53 (4.6)	5.70 (4.8)	3.75 (3.9)	<0.001
Peritraumatic Pain	1837 (87.6%)	446 (96.1%)	707 (91.0%)	402 (88.9%)	91 (50.3%)	<0.001	190 (91.3%)	575 (91.4%)	784 (84.5%)	<0.001	186 (96.4%)	91 (89.2%)	752 (90.4%)	556 (81.8%)	<0.001
Peritraumatic somatic symptoms^a^	5.17 (3.3)	6.29 (3.3)	5.31 (3.2)	4.62 (3.2)	3.71 (2.8)	<0.001	6.66 (3.1)	6.02 (3.3)	4.31 (3.0)	<0.001	6.65 (3.3)	5.24 (3.0)	5.80 (3.3)	4.09 (3.0)	<0.001
Week 8 Pain	894 (42.6%)	317 (68.3%)	396 (51.0%)	115 (25.4%)	30 (16.6%)	<0.001	137 (65.9%)	357 (56.8%)	350 (37.7%)	<0.001	141 (73.1%)	54 (52.9%)	443 (53.2%)	210 (30.9%)	<0.001
Week 8 Post-traumatic stress symptoms	28.22 (19.3)	41.17 (19.3)	28.84 (17.5)	19.33 (16.2)	16.72 (13.3)	<0.001	52.52 (16.2)	36.60 (15.3)	17.36 (13.5)	<0.001	52.81 (17.1)	23.84 (13.4)	34.98 (15.2)	13.68 (11.7)	<0.001
Week 8 Post-traumatic somatic symptoms^a^	7.72 (3.9)	9.75 (2.6)	8.32 (3.6)	5.75 (3.9)	5.15 (3.5)	<0.001	10.23 (2.6)	9.34 (3.0)	6.16 (3.8)	<0.001	10.21 (2.7)	7.49 (3.5)	9.05 (3.3)	5.46 (3.7)	<0.001
Week 8 Anxiety symptoms	7.23 (4.6)	9.68 (4.4)	7.22 (4.3)	5.56 (4.4)	5.42 (4.3)	<0.001	12.36 (3.3)	8.96 (3.9)	4.92 (3.9)	<0.001	11.93 (3.8)	7.09 (4.2)	8.37 (4.0)	4.44 (3.8)	<0.001
Week 8 Depressive Symptoms	54.36 (10.9)	59.85 (10.7)	54.46 (10.1)	50.81 (10.6)	49.41 (9.4)	<0.001	68.15 (7.8)	59.50 (7.8)	47.78 (8.1)	<0.001	64.94 (10.1)	52.88 (8.9)	57.17 (9.2)	47.91 (9.2)	<0.001

^a^Somatic symptoms are often referred to as “post-concussive symptoms”

↓: decrease, ↓↓: major decrease, ↑: increase

### Post-traumatic symptom measurement and trajectory models

Measurement models provided a good fit for all the ten latent constructs and all of them, except Anxiety, lacked strong measurement invariance over time (Supplementary Table 2). Due to the large sample size, many parameters were statistically different over time, but this represented a small absolute (<5%) and was therefore considered acceptable. Piecewise linear trajectory provided the best fit for all constructs except Anxiety whose trajectory follows a single linear pattern over time (Supplementary Table 3). The vast majority of the intercepts, slope factors, and their variances were significantly different from zero and the correlations between intercept and slopes were either small or non-significant (Supplementary Table 7).

**Fig. 1:**
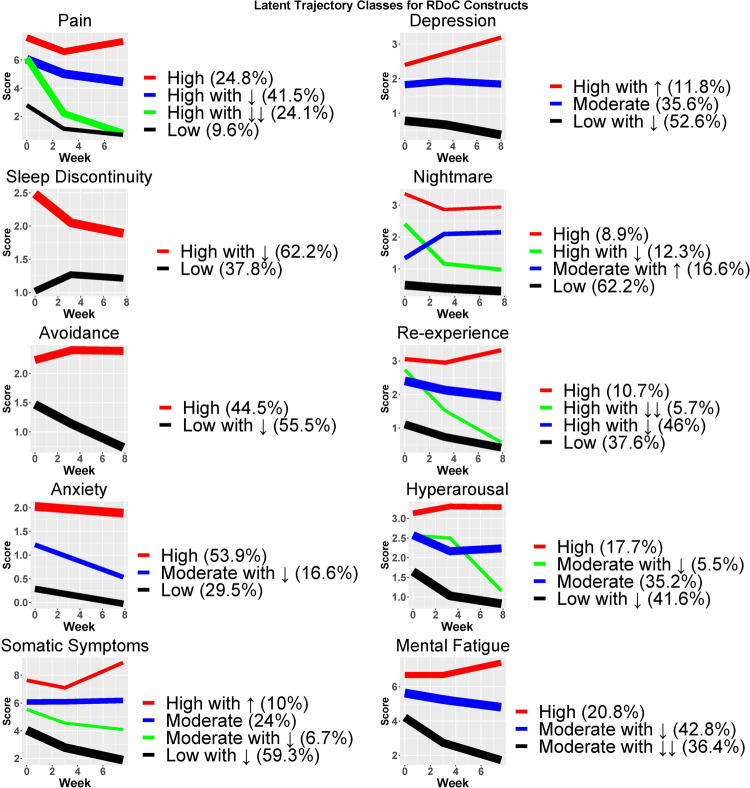
Common posttraumatic symptom trajectories during the eight weeks after motor vehicle collision (MVC). Common trajectories within each symptom domain were identified using latent growth curve mixture models developed from measurement models created using serial assessments of each symptom domain. (Each domain assessed six times in the first eight weeks after MVC.) Percentages within the legend for each symptom domain represent the percentage of trauma survivors within each latent class. The thickness of the lines represents the proportion of participants in each latent cluster. ↓: decreasing over time, ↓↓: major decrease over time, ↑: increasing over time.

Growth mixture models were used to identify common posttraumatic symptom trajectories (latent classes) during the eight weeks after MVC for pain, depression, sleep discontinuity, nightmares, avoidance, re-experiencing, anxiety, hyperarousal, mental/fatigue, and somatic symptoms (Fig. 1). Latent classes for each of these ten posttraumatic symptom trajectories, and associations between class membership and pre-trauma factors, peritraumatic symptoms, and traditional post-traumatic outcomes, are described briefly below and presented in detail in Tables 1–3.

**Table 2:** Characteristics of the overall sample and of individuals with specific symptom trajectories.

		Hyperarousal					Nightmare					Somatic Symptoms				
	Overall (*n* = 2097)	High (*n* = 312)	Moderate with ↓ (n = 98)	Moderate (*n* = 621)	Low with ↓ (*n* = 734)	*P*-value	High (*n* = 160)	High with ↓ (*n* = 221)	Moderate with ↑ (*n* = 299)	Low (*n* = 1120)	*P*-value	High with ↑ (*n* = 188)	Moderate (*n* = 449)	Moderate with ↓ (*n* = 125)	Low with ↓ (*n* = 1109)	*P*-value
Age	34.60 (12.7)	34.95 (12.0)	33.60 (12.2)	34.88 (12.9)	34.59 (12.8)	0.69	33.88 (11.7)	33.52 (11.0)	33.72 (12.4)	34.97 (13.0)	0.53	35.17 (12.1)	33.76 (12.6)	34.84 (12.8)	34.50 (12.7)	0.38
Sex at birth (Male)	760 (36.2%)	74 (23.7%)	24 (24.5%)	185 (29.8%)	329 (44.8%)	<0.001	48 (30.0%)	76 (34.4%)	103 (34.4%)	406 (36.2%)	0.46	55 (29.3%)	133 (29.6%)	43 (34.4%)	432 (39.0%)	0.001
Race/ethnicity						0.79					0.34					<0.001
Hispanic	257 (12.3%)	43 (13.8%)	13 (13.3%)	79 (12.7%)	86 (11.7%)		26 (16.2%)	28 (12.7%)	45 (15.1%)	130 (11.6%)		29 (15.4%)	59 (13.1%)	19 (15.2%)	121 (10.9%)	
Non-Hispanic White	647 (30.9%)	86 (27.6%)	29 (29.6%)	188 (30.3%)	246 (33.5%)		42 (26.2%)	72 (32.6%)	78 (26.1%)	365 (32.6%)		38 (20.2%)	112 (24.9%)	38 (30.4%)	400 (36.1%)	
Non-Hispanic Black	1102 (52.6%)	171 (54.8%)	52 (53.1%)	325 (52.3%)	372 (50.7%)		86 (53.8%)	112 (50.7%)	159 (53.2%)	580 (51.8%)		112 (59.6%)	262 (58.4%)	61 (48.8%)	537 (48.4%)	
Non-Hispanic Other	81 (3.9%)	9 (2.9%)	3 (3.1%)	26 (4.2%)	28 (3.8%)		4 (2.5%)	8 (3.6%)	14 (4.7%)	42 (3.8%)		7 (3.7%)	13 (2.9%)	7 (5.6%)	46 (4.1%)	
Education (High School or Less)	689 (32.9%)	117 (37.5%)	33 (33.7%)	201 (32.4%)	223 (30.4%)	0.16	68 (42.5%)	68 (30.8%)	114 (38.1%)	341 (30.4%)	0.003	80 (42.6%)	165 (36.7%)	50 (40.0%)	312 (28.1%)	<0.001
Income (<=$35,000)	981 (46.8%)	179 (57.4%)	60 (61.2%)	322 (51.9%)	342 (46.6%)	0.003	93 (58.1%)	119 (53.8%)	177 (59.2%)	522 (46.6%)	<0.001	114 (60.6%)	258 (57.5%)	76 (60.8%)	487 (43.9%)	<0.001
Pre-Trauma Pain	662 (31.6%)	141 (45.2%)	27 (27.6%)	222 (35.7%)	176 (24.0%)	<0.001	83 (51.9%)	77 (34.8%)	126 (42.1%)	292 (26.1%)	<0.001	84 (44.7%)	184 (41.0%)	51 (40.8%)	273 (24.6%)	<0.001
Pre-Trauma somatic symptoms^a^	3.90 (3.5)	5.31 (4.0)	3.52 (3.6)	4.61 (3.6)	2.98 (3.0)	<0.001	5.90 (4.3)	5.02 (3.9)	4.77 (3.7)	3.30 (3.1)	<0.001	5.27 (4.3)	4.74 (3.8)	4.39 (3.8)	3.38 (3.1)	<0.001
Pre-Trauma Depression	48.31 (10.7)	52.72 (12.4)	48.21 (9.5)	49.39 (10.8)	45.97 (9.3)	<0.001	55.02 (13.7)	50.64 (11.3)	50.51 (10.7)	46.63 (9.6)	<0.001	51.27 (12.7)	49.65 (10.9)	50.18 (11.0)	47.32 (10.0)	<0.001
Pre-Trauma Anxiety symptoms	5.02 (4.7)	7.33 (5.3)	4.90 (4.2)	5.61 (4.6)	3.71 (3.9)	<0.001	7.48 (5.6)	6.37 (5.0)	5.94 (4.6)	4.31 (4.3)	<0.001	6.09 (5.2)	5.66 (4.9)	5.66 (4.6)	4.64 (4.5)	<0.001
Peri-traumatic Pain	1837 (87.6%)	289 (92.6%)	89 (90.8%)	562 (90.5%)	609 (83.0%)	<0.001	151 (94.4%)	204 (92.3%)	277 (92.6%)	947 (84.6%)	<0.001	177 (94.1%)	413 (92.0%)	112 (89.6%)	935 (84.3%)	<0.001
Peritraumatic somatic symptoms^a^	5.17 (3.3)	6.58 (3.3)	5.22 (3.1)	5.79 (3.2)	4.11 (3.0)	<0.001	7.52 (3.2)	6.02 (3.3)	6.05 (3.2)	4.52 (3.1)	<0.001	6.93 (3.3)	5.88 (3.2)	5.86 (3.4)	4.56 (3.1)	<0.001
Week 8 Pain	894 (42.6%)	204 (65.4%)	62 (63.3%)	336 (54.1%)	242 (33.0%)	<0.001	112 (70.0%)	112 (50.7%)	183 (61.2%)	440 (39.3%)	<0.001	121 (64.4%)	259 (57.7%)	81 (64.8%)	404 (36.4%)	<0.001
Week 8 Post-traumatic stress symptoms	28.22 (19.3)	49.86 (16.3)	26.70 (14.3)	33.25 (14.9)	14.98 (12.9)	<0.001	54.38 (16.6)	31.84 (16.5)	40.25 (16.6)	20.32 (14.9)	<0.001	48.56 (19.3)	35.70 (17.9)	33.54 (15.3)	21.22 (16.3)	<0.001
Week 8 Post-traumatic somatic symptoms^a^	7.72 (3.9)	10.10 (2.7)	7.90 (3.4)	9.00 (3.1)	5.60 (3.8)	<0.001	10.64 (2.5)	8.73 (3.5)	9.70 (3.3)	6.59 (3.8)	<0.001	10.93 (2.0)	9.66 (3.3)	9.50 (3.2)	6.15 (3.6)	<0.001
Week 8 Anxiety symptoms	7.23 (4.6)	11.64 (3.6)	7.04 (4.1)	8.07 (3.9)	4.58 (3.9)	<0.001	11.87 (4.0)	8.13 (4.3)	9.11 (4.2)	5.84 (4.2)	<0.001	10.66 (4.3)	8.32 (4.5)	8.16 (4.3)	6.07 (4.4)	<0.001
Week 8 Depressive Symptoms	54.36 (10.9)	64.06 (9.8)	52.78 (8.7)	56.48 (8.9)	48.52 (9.4)	<0.001	66.85 (9.0)	55.87 (9.5)	60.08 (9.2)	50.61 (9.6)	<0.001	64.15 (10.1)	57.42 (9.6)	56.02 (9.7)	51.20 (10.2)	<0.001

^a^Somatic symptoms are often referred to as “post-concussive symptoms”

↓: decrease, ↑: increase

**Table 3:** Characteristics of the overall sample and of individuals with specific symptom trajectories.

		Anxiety				Avoidance				Mental Fatigue				Sleep Discontinuity	
	Overall (*n* = 2097)	High (n = 952)	Moderate with ↓ (*n* = 292)	Low (*n* = 521)	*P*-value	High (*n* = 805)	Low with ↓ (*n* = 1002)	*P*-value	High (*n* = 390)	Moderate with ↓ (*n* = 800)	Moderate with ↓ ↓ (*n* = 681)	*P*-value	High with ↓ (*n* = 1119)	Low (*n* = 681)	*P*-value
Age	34.60 (12.7)	34.26 (12.4)	36.01 (12.5)	34.78 (13.3)	0.065	35.38 (12.7)	33.90 (12.6)	0.007	36.64 (13.1)	34.53 (13.1)	32.99 (11.5)	<0.001	35.29 (12.6)	33.17 (12.4)	<0.001
Sex at birth (Male)	760 (36.2%)	274 (28.8%)	89 (30.5%)	249 (47.8%)	<0.001	233 (28.9%)	400 (39.9%)	<0.001	111 (28.5%)	260 (32.5%)	292 (42.9%)	<0.001	372 (33.2%)	261 (38.3%)	0.03
Race/ethnicity					0.24			<0.001				0.22			0.44
Hispanic	257 (12.3%)	133 (14.0%)	33 (11.3%)	55 (10.6%)		115 (14.3%)	111 (11.1%)		48 (12.3%)	110 (13.8%)	70 (10.3%)		152 (13.6%)	77 (11.3%)	
Non-Hispanic White	647 (30.9%)	304 (31.9%)	88 (30.1%)	157 (30.1%)		202 (25.1%)	359 (35.8%)		118 (30.3%)	241 (30.1%)	229 (33.6%)		349 (31.2%)	208 (30.5%)	
Non-Hispanic Black	1102 (52.6%)	477 (50.1%)	161 (55.1%)	282 (54.1%)		459 (57.0%)	483 (48.2%)		209 (53.6%)	417 (52.1%)	346 (50.8%)		571 (51.0%)	366 (53.7%)	
Non-Hispanic Other	81 (3.9%)	32 (3.4%)	9 (3.1%)	25 (4.8%)		24 (3.0%)	45 (4.5%)		12 (3.1%)	28 (3.5%)	33 (4.8%)		40 (3.6%)	28 (4.1%)	
Education (High School or Less)	689 (32.9%)	327 (34.3%)	90 (30.8%)	157 (30.1%)	0.20	288 (35.8%)	304 (30.3%)	0.016	132 (33.8%)	263 (32.9%)	212 (31.1%)	0.62	372 (33.2%)	219 (32.2%)	0.67
Income (<=$35,000)	981 (46.8%)	504 (52.9%)	170 (58.2%)	229 (44.0%)	0.001	459 (57.0%)	455 (45.4%)	<0.001	229 (58.7%)	399 (49.9%)	307 (45.1%)	<0.001	584 (52.2%)	327 (48.0%)	0.28
Pre-Trauma Pain	662 (31.6%)	386 (40.5%)	80 (27.4%)	100 (19.2%)	<0.001	330 (41.0%)	253 (25.2%)	<0.001	183 (46.9%)	279 (34.9%)	130 (19.1%)	<0.001	412 (36.8%)	166 (24.4%)	<0.001
Pre-Trauma Somatic Symptoms^a^	3.90 (3.5)	5.10 (3.7)	3.38 (3.1)	2.32 (2.6)	<0.001	4.71 (3.8)	3.39 (3.2)	<0.001	5.45 (3.9)	4.43 (3.5)	2.56 (2.8)	<0.001	4.55 (3.7)	3.05 (3.2)	<0.001
Pre-Trauma Depression	48.31 (10.7)	51.60 (11.4)	46.80 (8.8)	43.75 (8.3)	<0.001	50.57 (11.5)	46.91 (9.8)	<0.001	51.98 (12.3)	49.71 (10.3)	44.99 (9.0)	<0.001	49.74 (11.2)	46.47 (9.6)	<0.001
Pre-Trauma Anxiety symptoms	5.02 (4.7)	6.59 (4.9)	4.13 (3.8)	2.85 (3.3)	<0.001	6.03 (5.0)	4.36 (4.3)	<0.001	6.84 (5.2)	5.61 (4.6)	3.50 (3.9)	<0.001	5.70 (4.8)	4.15 (4.3)	<0.001
Peritraumatic Pain	1837 (87.6%)	867 (91.1%)	253 (86.6%)	429 (82.3%)	<0.001	742 (92.2%)	843 (84.1%)	<0.001	369 (94.6%)	724 (90.5%)	544 (79.9%)	<0.001	1028 (91.9%)	551 (80.9%)	<0.001
Peritraumatic Somatic Symptoms^a^	5.17 (3.3)	6.16 (3.2)	4.80 (3.0)	3.66 (2.9)	<0.001	5.99 (3.3)	4.59 (3.1)	<0.001	6.85 (3.1)	5.46 (3.2)	3.96 (3.0)	<0.001	5.88 (3.2)	4.16 (3.1)	<0.001
Week 8 Pain	894 (42.6%)	546 (57.4%)	158 (54.1%)	140 (26.9%)	<0.001	489 (60.7%)	359 (35.8%)	<0.001	274 (70.3%)	407 (50.9%)	184 (27.0%)	<0.001	620 (55.4%)	227 (33.3%)	<0.001
Week 8 Post-traumatic stress symptoms	28.22 (19.3)	39.85 (17.1)	22.83 (12.2)	11.20 (10.1)	<0.001	39.74 (17.6)	18.71 (14.9)	<0.001	45.32 (18.1)	30.80 (15.8)	15.00 (13.1)	<0.001	33.28 (19.1)	19.45 (16.2)	<0.001
Week 8 Somatic Symptoms^a^	7.72 (3.9)	9.57 (2.9)	7.41 (3.5)	4.70 (3.6)	<0.001	9.36 (3.2)	6.38 (3.9)	<0.001	10.20 (2.4)	8.78 (3.3)	5.01 (3.6)	<0.001	8.65 (3.5)	6.17 (4.0)	<0.001
Week 8 Anxiety symptoms	7.23 (4.6)	9.68 (4.1)	6.50 (3.7)	3.28 (3.1)	<0.001	9.31 (4.3)	5.47 (4.2)	<0.001	10.72 (4.1)	7.60 (4.1)	4.68 (4.0)	<0.001	8.22 (4.5)	5.50 (4.4)	<0.001
Week 8 Depression	54.36 (10.9)	60.09 (9.4)	52.46 (8.4)	45.16 (7.7)	<0.001	59.18 (10.2)	50.24 (9.7)	<0.001	62.39 (10.1)	55.69 (9.2)	47.88 (9.2)	<0.001	56.79 (10.5)	50.09 (10.3)	<0.001

^a^Somatic symptoms are often referred to as “post-concussive symptoms”

↓: decrease

#### Pain

Four trajectories (classes) of post-traumatic pain were identified: high (25%), high initial with some recovery (41%), high initial with marked recovery (10%), and low (10%) (Fig. 1). A higher post-traumatic pain trajectory was associated with socioeconomic disadvantage, Non-Hispanic Black ethnicity/race, and a greater burden of somatic, depressive, and anxiety/posttraumatic stress symptoms both prior to trauma and after trauma (Table 1).

#### Depression

Three trajectories of post-traumatic depressive symptoms (feeling worthless, sad, trouble experiencing positive feelings) were identified: high and increasing over time (12%), persistent moderate (36%), and low and decreasing over time (52%) (Fig. 1). A greater burden of post-traumatic depressive symptoms was associated with socioeconomic disadvantage, younger age, and a greater burden of pain, somatic, and anxiety/posttraumatic stress symptoms both prior to trauma and after trauma (Table 1).

#### Re-experiencing

Four post-traumatic trajectories of re-experiencing symptoms (unwanted memories or feeling upset or strong physical reactions to trauma reminders) were identified: high (11%), high initial with some improvement over time (46%), high initial with marked improvement over time (6%), and low initial and decreasing (37%) (Fig. 1). A high post-traumatic re-experiencing symptom burden were associated with Non-Hispanic Black ethnicity/race, greater socioeconomic disadvantage, and a greater burden of pain, somatic, depressive, and anxiety/posttraumatic stress symptoms both prior to trauma and after trauma (Table 1).

#### Hyperarousal

Four trajectories of post-traumatic hyperarousal symptoms (feeling superalert, watchful, on guard, or jumpy/easily startled) were identified: high (18%) initial moderate with the marked decline (5%), moderate (35%), and low initial with the decline (36%) (Fig. 1). A high post-traumatic hyperarousal symptom trajectory was associated with female sex and a greater burden of pain, somatic, depressive, and anxiety/posttraumatic stress symptoms both prior to trauma and after MVC (Table 2).

#### Nightmares

Four post-traumatic trajectories of nightmare symptoms were identified: high (9%), high with the rapid decline (12%), moderate and increasing (17%), and low (62%) (Fig. 1). Individuals with a high or moderate/increasing burden of post-traumatic nightmares had the lowest education and income. A greater burden of post-traumatic nightmares was also associated with a greater burden of pain, somatic, depressive, and anxiety/posttraumatic stress symptoms both prior to trauma and after trauma (Table 2).

#### Somatic symptoms

Four post-traumatic trajectories of somatic symptoms (headaches, dizziness, and nausea) were identified: high initial and increasing (10%), moderate (24%), initial moderate with improvement (7%), and low with improvement (59%) (Fig. 1). A higher post-traumatic somatic symptom trajectory was associated with a greater burden of pain, somatic, depressive, and anxiety/posttraumatic stress symptoms both prior to trauma and after trauma (Table 2).

#### Anxiety

Three post-traumatic symptom trajectories of anxiety were identified: high (54%), moderate initial with decrease over time (17%), and low (29%) (Fig. 1). A high post-traumatic anxiety symptom trajectory was associated with a greater burden of pain, somatic, depressive, and anxiety/posttraumatic stress symptoms both prior to trauma and after the MVC (Table 3).

#### Avoidance

Two post-traumatic trajectories of avoidance (avoiding memories, thoughts, feelings, or reminders of the MVC) were identified: high avoidance symptoms across time (45%) and low initial avoidance symptoms with continued improvement (55%) (Fig. 1). A high burden of post-traumatic avoidance symptoms was associated with socioeconomic disadvantage, female sex, and Non-Hispanic Black ethnicity/race. A high burden of post-traumatic avoidance symptoms was also associated with a greater burden of pain, somatic, depressive, and anxiety/posttraumatic stress symptoms both prior to trauma and after trauma (Table 3).

#### Mental/fatigue

Three trajectories of mental/fatigue symptoms (difficulty with concentration, thinking, and fatigue) were identified: high initial and increasing (21%), moderate initial with some decrease over time (43%), and moderate initial with a marked decrease (36%) (Fig. 1). A greater post-traumatic burden of difficulty with concentration, thinking, and fatigue was associated with relatively older age, female sex at birth, lower income, and a greater burden of pain, somatic, depressive, and anxiety/posttraumatic stress symptoms prior to trauma and over time (Table 3).

#### Sleep discontinuity

Two trajectories of sleep discontinuity (problems falling asleep, staying asleep, and/or waking too early) were identified: high initial sleep discontinuity, which improved most during the first two weeks after MVC and then more slowly (62%), and low sleep discontinuity (38%) (Fig. 1). Higher post-traumatic sleep discontinuity was associated with relatively older age, female sex, and a greater burden of pain, somatic, depressive, and anxiety/posttraumatic stress symptoms both prior to trauma and after trauma (Table 3).

### Correlations between initial post-traumatic symptoms across domains

**Fig. 2:**
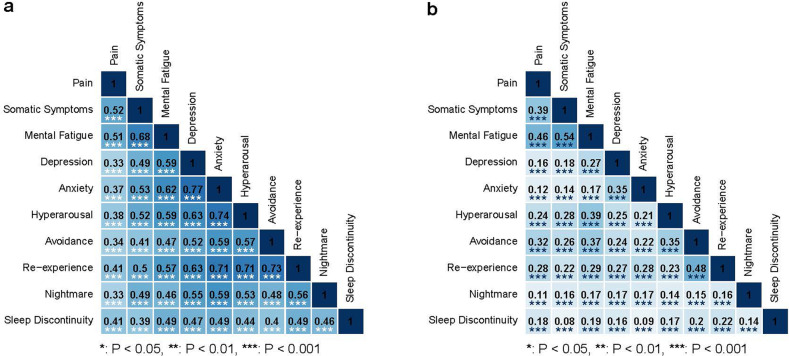
Correlations among common post-traumatic symptoms during the eight weeks after motor vehicle collision (MVC). Correlations among intercepts **a** and slopes **b** of the trajectories of common post-traumatic symptoms during the eight weeks after motor vehicle collision (MVC) are displayed; darker shading indicates a higher correlation. Trajectory intercepts from latent growth curve models represent peritraumatic symptom levels in the initial days after MVC. Slopes represent changes in symptoms during the first 8 weeks after trauma.

Post-traumatic trajectory intercepts represent peritraumatic symptom levels in the initial days after trauma. Timing of initial symptom assessments according to the domain is shown in Supplementary Table 1; all initial symptoms were performed within five days of trauma. The great majority of associations between post-traumatic symptom intercepts were moderate to strong (Fig. 2a). The strongest peritraumatic associations between trajectory intercepts were observed across anxiety domains, between anxiety and depression, and between anxiety and hyperarousal (Fig. 2a). Symptom domain correlations between domains not within the same traditional diagnosis were often as strong/nearly as strong as correlations between domains within a diagnosis (e.g., hyperarousal-somatic *r* = 0.52, hyperarousal-mental/fatigue *r* = 0.59, hyperarousal-avoidance *r* = 0.57, hyperarousal-nightmares 0.53). Similar patterns are observed for participants who reported hitting their heads and not hitting their heads during the MVC (Supplementary Fig. 1).

### Correlations between slopes

Symptom domain correlations were generally weaker for slopes than for intercepts (Fig. 2b), indicating that changes in symptoms over time were not as highly correlated as peritraumatic symptom levels in the days after trauma. This may also represent symptom domains regressing to the mean over time or variable patterns of recovery and persistence among the various symptoms. Moderate correlations in changes in symptom intensity over time were observed between mental/fatigue and somatic symptoms (*r* = 0.54, *p* < 0.001), mental/fatigue and pain (*r* = 0.46, *p* < 0.001), and re-experience and avoidance (*r* = 0.48, *p* < 0.001) (Fig. 2b). Similar patterns are observed for participants who reported hitting their heads and not hitting their heads during the MVC (Supplementary Fig. 2).

## Discussion

To our knowledge, these data represent the largest published longitudinal cohort of survivors of MVC, one of the most common life-threatening traumatic stress exposures in industrialized nations. Study participants were recruited at the time of ED presentation and assessed during the initial eight weeks after the MVC trauma. Analyses of frequent serial assessments of ten common post-traumatic symptom domains, including core symptom domains within traditional diagnoses of post-traumatic pain, stress, depression, and post-concussion syndrome, provided several valuable insights into post-traumatic symptom trajectories, inter-relationships between these trajectories, and shared and distinct vulnerability factors. First, these analyses demonstrate that a broad array of post-traumatic symptoms are common during the initial months after trauma. In addition, these analyses demonstrate that symptoms across different traditional post-traumatic diagnoses are substantially correlated with one another. These correlations are consistent with the highly integrated nature of the physiologic response to life threats, and the pleiotropic effects of stress system molecules activated as part of this response [38]. These findings suggest that individuals who present to specific clinics with a high burden of post-traumatic pain, posttraumatic stress, depressive symptoms, or somatic (“post-concussive”) symptoms should be screened for other morbidity and referred as appropriate.

These data also suggest that the care organization of trauma survivors into multidisciplinary clinics should be considered, to explore whether more comprehensive care yields better outcomes. For example, across a substantial proportion of participants, self-reported cognitive functioning worsened during the initial weeks after MVC. This finding may have implications for return to routine function (e.g., work), that could be more consistently/effectively managed by a multidisciplinary clinic. Similarly, multidimensional diagnoses that categorize patients across the full range of post-traumatic symptoms experienced by a trauma survivor should be explored, to evaluate if such categorizations have any advantage over current diagnoses in treatment selection or outcome prediction. For example, a patient with poor sleep continuity, high depression, high anxiety, and high pain, might require a different treatment approach than a patient with high depression, high anxiety, but low pain, and normal sleep continuity.

Another overarching finding from these analyses is that, while some subgroups of individuals with specific post-traumatic symptoms experience marked changes in the initial months after trauma, peritraumatic symptom severity is in general a dominant factor influencing outcome/identifying vulnerability. Individuals with the highest burden of peritraumatic symptoms in the days after trauma generally recover the least in terms of those symptoms. These data suggest that individuals at high risk of prolonged suffering with specific symptoms may be relatively easy to identify for secondary preventive interventions, from a high burden of such symptoms in the days after trauma. Post-traumatic neuropsychiatric sequelae cannot be prevented today, in the same way that post-traumatic wound infection could not be prevented two centuries ago. In part, this is because few studies have tested secondary preventive interventions in the early aftermath of trauma, but these data suggest that such individuals are readily identifiable.

The sociodemographic disadvantage was a risk factor for a worse recovery trajectory across the great majority of post-traumatic symptoms. As noted above, our study sample was comprised of a diverse, majority Black sample recruited from more than 25 ED urban ED. This is a historically understudied population in longitudinal trauma survivor studies [39]. Rates of pre-MVC morbidity were relatively high, and post-traumatic APNS was common: nearly 73% of the individuals in the sample had one or more post-traumatic symptom trajectories categorized as high or increasing. These data highlight the substantial burden of APNS experienced by vulnerable populations who present to US EDs after common trauma exposures such as MVC.

Of note, we recognize that there are a great many specific relationships that investigators may wish to explore, based on their own interests, between specific sociodemographic, pre-trauma, and/or peritraumatic characteristics, and/or between an individual or multiple post-traumatic symptom trajectories and traditional post-traumatic outcomes. To facilitate this, we have created an interactive data visualization tool to explore the data: http://itr.med.unc.edu/aurora/parcoord/. We encourage readers to use this tool to gain additional insights regarding relationships of interest.

This study had a number of strengths, including large sample size, frequent assessment of indicator variables (scale of days as opposed to weeks) to create relatively detailed trajectories, use of measurement models with a good fit to develop latent growth curves and mixture models, and a diverse understudied sample. Our study had specific limitations which should be considered when interpreting our results. First, the study was limited to individuals experiencing MVC. While MVC is one of the most common life-threatening trauma exposures in industrialized countries, post-traumatic symptom trajectories and their relationships after other types of trauma exposure (e.g., sexual assault) are not known. In addition, approximately 95% of patients enrolled in this study were MVC patients who were seen in the ED and discharged to home after evaluation. However, these data are consistent with national data: the overwhelming majority of individuals who present to the ED after MVC are not admitted to the hospital [40]. Our work is formative in understanding post-traumatic sequelae and external validation is warranted to test the generalizability of our model. Third, while there is growing interest in biobehavioral markers of specific post-traumatic trajectories, this analysis focused on self-report symptoms alone. However, such symptoms are the sine qua non of the common post-traumatic symptom domains reported by trauma survivors across the globe and further work is needed to examine the clustering and patterns of symptoms that may occur within groups of individuals. Understanding single-symptom trajectories lay the groundwork to explore more dynamics of multiple symptoms over time. In addition, as with any other observational study, associations within the data cannot be interpreted causally (e.g., low income with somatic symptoms). Lastly, numerous modeling decisions (such as whether to include correlated error terms in tests of longitudinal invariance) were made post-hoc and impact the interpretation of change and correlated change over time, and limit the generalizability of these results to other cohorts.

In summary, we used brief serial self-report surveys to characterize post-traumatic symptom trajectories across a broad range of mental and physical health symptoms in several thousand trauma survivors. Substantial post-traumatic symptoms were common across domains, and post-traumatic symptoms showed moderate to high correlation with one another, both across as well as within traditional diagnoses. These results suggest that any individual with substantial symptoms in one domain, or any post-traumatic outcome (e.g., posttraumatic stress), should be screened for others and that individual post-traumatic diagnoses capture only a part of the survivor experience. In addition, across symptom types, peritraumatic symptom severity appears to be a dominant factor identifying vulnerability to poor recovery. Investigators interested in specific relationships of interest between individual, peritraumatic, and recovery characteristics are encouraged to explore the data further with our interactive data visualization tool.

## Supplementary information

Supplemental Material
