# Supplementary material for: Use of serial smartphone-based assessments to characterize diverse neuropsychiatric symptom trajectories in a large trauma survivor cohort
Source: Transl Psychiatry. 2023 Jan 7;13:4. doi: 10.1038/s41398-022-02289-y (PMC9823011; doi:10.1038/s41398-022-02289-y)
Supplement: Supplementary file 1 — Supplemental Material [file 41398_2022_2289_MOESM1_ESM.docx]

**SUPPLEMENTARY STISTICAL ANALYSES**

For each latent construct, the analysis includes four steps: (1) identification of an appropriate structure for the base cross-time point measurement model using confirmatory factor analysis; (2) checking measurement invariance for the measurement model across time using likelihood tests; (3) using a variety of functional forms over time, estimate and compare trajectory models using growth curve models constructed using the time-specific factors for the measurement model embedded; (4) estimation and identification of latent trajectory classes using growth mixture models. Analyses were conducted using Mplus version 8.

### **Determination of Base Measurement Models**

All these latent constructs are measured 6 time points within 8 weeks after enrollment. A joint measurement model that includes all time points is used to define each latent construct and to study the relationship of the time-specific latent construct measurement models measured at different time points. The temporal correlations of a given indicator variable were generally not fully explained by this joint model. Temporal correlations of error term of same indicator variables at adjacent time points or all time points are introduced to account for the autocorrelations. Model fit indices, such as Comparative Fit Index (CFI), Tucker-Lewis Index (TLI), Root Mean Square Error of Approximation (RMSEA) and Standardized Root Mean Square Residual (SRMR), are used to evaluate the fit of the measurement model (Supplement Table 2).

### **Measurement Invariance**

In order to build trajectory models using the time-specific factors, the consistency of the measurement model needs to be checked and established. Otherwise, the latent constructs defined by the measurement model are not comparable across time. All the measurement models are evaluated for strong (or scalar) invariance across time. Strong measurement invariance means that (1) the factor loading structure underlying these indicator variables are the same; (2) the factor loading parameters are equivalent; (3) the intercept (or mean) parameters of indicator variables are the same. Measurement invariance are tested using likelihood ratio test. It is not uncommon that some of the model parameters may not satisfy measurement invariance, especially when sample size is large. For example, the intercept parameter of indicator variables may be invariant across time except the first time point. If only a few of the time points are not invariant, the differences are less than 5%, and they can be explained by substantive reasons, measurement model with partial invariance are used for the rest of the analyses.

### **Estimation of Trajectory Models using Growth Curve Model, Determining Best Functional Form**

The full sample growth trajectory pattern of each latent construct across the first 8 weeks after enrollment were modeled by growth curve models for the time-specific factors of the embedded measurement model. Different trajectory patterns across time, such as linear and piecewise, were explored and the best trajectory pattern for each latent construct was selected based on log likelihood ratio test.

### **Identify Latent Trajectory Classes using Growth Mixture Models**

Based on the best functional form identified, latent trajectory classes were estimated with that form using growth mixture models. Number of latent class was determined by model fit, BIC, percentage of participants in each latent class and clinical expectations. Models with convergence issues or has latent classes with less than 5% participants are excluded first. Afterwards, BIC is used to select the best model. For models with similar BICs, clinical expectations are used to guide the final model selection. Model output was then used to classify participants into different latent trajectory classes. Trajectory patterns are different across latent classes with respect to either or both the intercept and slopes. In order to successfully estimate these models a variety of modeling strategies were employed including constraining variance parameters to be positive and correlation parameter estimates to be less than or equal to 1.0, and setting small negative variances that were non-significant to zero. Sometimes the measurement error correlation structure had to be simplified.

**SUPPLEMENTARY TABLES AND FIGURES**

**Supplementary Table 1. Latent constructs indicator variable questions from smartphone-based follow-up surveys**

| **Latent Constructs** | **Timepoints (first 8 weeks)** | **Questions** | **Response options** |
| --- | --- | --- | --- |
| **Pain** | Days: 1,9,21,31,43,53 | 1. How would you rate your pain in the past 24 hours at its worst? 2. How would you rate your pain in the past 24 hours on average? | No pain Severe pain  0 1 2 3 4 5 6 7 8 9 10 |
| **Depression** | Days: 5,19,29,39,51,61 | 1. Over the past 24 hours, how often did you feel down on yourself, no good, or worthless? 2. Over the past 24 hours, how often did you feel sad depressed, or empty? 3. Over the past 24 hours, how often did you have trouble experiencing positive feelings? (for example, being unable to feel happiness or having loving feelings for people close to you) | Never Rarely Sometimes Often Very often  [0] [1] [2] [3] [4] |
| **Sleep Discontinuity** | Days: 3,15,25,35,47,57 | 1. Over the last few nights, how much of a problem have you had falling asleep? 2. Over the last few nights, how much of a problem have you had staying asleep all night? 3. Over the last few nights, how much of a problem have you had waking up too early in the morning? | None A little Some A lot Extremely  [0] [1] [2] [3] [4] |
| **Nightmare** | Days: 3,15,25,35,47,57 | 1. Over the last few nights, how much of a problem have you had with nightmares or bad dreams about the event? 2. Over the last few nights, how much of a problem have you had with nightmares or bad dreams about other things? 3. Over the last few nights, how much of a problem have you had with panic attacks during the night? | None A little Some A lot Extremely  [0] [1] [2] [3] [4] |
| **Anxiety** | Days: 5,18,28,38,50,60 | 1. Over the past 24 hours, how often did you have severe anxiety or panic? 2. Over the past 24 hours, how often did you feel very nervous, worried, or anxious? | Never Rarely Sometimes Often Very often  [0] [1] [2] [3] [4] |
| **Hyperarousal** | Days: 5,18,28,38,50,60 | 1. Over the past 24 hours, how often were you “superalert” or watchful, or on guard? 2. Over the past 24 hours, how often did you feel jumpy or easily startled? | Never Rarely Sometimes Often Very often  [0] [1] [2] [3] [4] |
| **Avoidance** | Days: 4,17,27,37,49,59 | 1. Over the past 24 hours, how often did you avoid memories, thoughts, or feelings related to the event? 2. Over the past 24 hours, how often did you avoid external reminders of the event? (e.g., people, places, conversations, or activities) | Never Rarely Sometimes Often Very often  [0] [1] [2] [3] [4] |
| **Re-experience** | Days: 4,17,27,37,49,59 | 1. Over the past 24 hours, how often did you have repeated, disturbing, and unwanted memories of the event? 2. Over the past 24 hours, how often did you feel very upset when something reminded you of the event? 3. Over the past 24 hours, how often did you have strong physical reactions when something reminded you of the event, like heart pounding, trouble breathing, or sweating? | Never Rarely Sometimes Often Very often  [0] [1] [2] [3] [4] |
| **Somatic Symptoms** | Days: 2,11,23,33,45,55 | 1. Over the past 24 hours, how much of a problem have you had with headaches? 2. Over the past 24 hours, how much of a problem have you had with dizziness? 3. Over the past 24 hours, how much of a problem have you had with nausea? | No problem Severe problem  0 1 2 3 4 5 6 7 8 9 10 |
| **Mental**  **Fatigue** | Days: 2,11,23,33,45,55 | 1. Over the past 24 hours, how much of a problem have you had with fatigue? 2. Over the past 24 hours, how much of a problem have you had concentrating? 3. Over the past 24 hours, how much of a problem have you had taking longer to think? | No problem Severe problem  0 1 2 3 4 5 6 7 8 9 10 |

**Supplementary Table 2.** Measurement Model Fit, Temporal Correlations, and Measurement Invariance. For temporal correlation, None means no extra correlation of error teams, and Adjacent means correlation of error terms of same indicator variable at adjacent time points. For measurement invariance, Full means full measurement invariance, and Partial means partial measurement invariance.

| **Latent Construct** | **Temporal**  **Correlation** | **CFI** | **TLI** | **SRMR** | **RMSEA** | **Measurement Invariance** |
| --- | --- | --- | --- | --- | --- | --- |
| Pain | None | 0.979 | 0.964 | 0.007 | 0.077 | Partial |
| Depression | None | 0.952 | 0.938 | 0.028 | 0.069 | Partial |
| Sleep Discontinuity | Adjacent | 0.942 | 0.916 | 0.042 | 0.066 | Partial |
| Nightmare | Adjacent | 0.934 | 0.904 | 0.044 | 0.085 | Partial |
| Avoidance | None | 0.987 | 0.979 | 0.013 | 0.042 | Partial |
| Re-experience | None | 0.932 | 0.914 | 0.033 | 0.074 | Partial |
| Anxiety | None | 0.919 | 0.864 | 0.036 | 0.111 | Full |
| Hyperarousal | Adjacent | 0.947 | 0.880 | 0.038 | 0.102 | Partial |
| Somatic Symptoms | Adjacent | 0.920 | 0.883 | 0.049 | 0.089 | Partial |
| Mental Fatigue^1^ | None | 0.901 | 0.873 | 0.062 | 0.107 | Partial |

^1^MentalFatigue are measured by three questions related to concentration, thinking and fatigue.

**Supplementary Table 3. Selected trajectory** pattern, model fit indices and estimates of intercept and slope parameters of average trajectory models for each latent construct. For Growth Trajectory, Linear means linear trajectory model, and Piecewise means linear piecewise trajectory model.

| **Latent Construct** | **Growth Trajectory** | **CFI** | **TLI** | **SRMR** | **RMSEA** |
| --- | --- | --- | --- | --- | --- |
| Pain | Piecewise | 0.972 | 0.968 | 0.046 | **0.072** |
| Depression | Piecewise | 0.951 | 0.949 | 0.029 | **0.063** |
| Sleep Discontinuity | Piecewise | 0.941 | 0.931 | 0.044 | **0.059** |
| Nightmare | Piecewise | 0.932 | 0.920 | 0.047 | **0.072** |
| Avoidance | Piecewise | 0.985 | 0.984 | 0.025 | **0.037** |
| Re-experience | Piecewise | 0.932 | 0.930 | 0.035 | **0.067** |
| Anxiety | Linear | 0.917 | 0.916 | 0.041 | **0.087** |
| Hyperarousal | Piecewise | 0.946 | 0.923 | 0.042 | **0.082** |
| Somatic Symptoms | Piecewise | 0.918 | 0.904 | 0.051 | **0.081** |
| Mental Fatigue^1^ | Piecewise | 0.949 | 0.937 | 0.053 | **0.074** |

^1^Mental Fatigue are measured by three questions related to concentration, thinking and fatigue.

**Supplementary Table 4.** Loss to follow-up: Correlations of main outcomes (first column) and completion rate of four main activities (first row). Completion rate was defined as the proportion of finished tasks over the number of the tasks had become available to the participant.

|  | **Survey** | **Watch Wearing** | **Neurocognitive Test** | **Flash Survey** |
| --- | --- | --- | --- | --- |
| Pre-Trauma Pain | .0169 | 0.037 | 0.011 | 0.011 |
| Pre-Trauma Depression | -.0167 | -0.007 | -0.021 | -0.011 |
| Pre-Trauma PTSD | -.0194 | -0.031 | -0.069** | -0.051* |
| Pre-Trauma Somatic | -.0284 | -0.014 | -0.020 | -0.022 |
| Peritraumatic Pain | -.0131 | -0.052 | -0.020 | -0.027 |
| Peritraumatic Somatic | -.0263 | -0.033 | -0.011 | -0.029 |
| Week 2 Pain | .051^*^ | .062^*^ | 0.032 | 0.046* |
| Week 2 Depression | .0095 | -0.009 | 0.012 | 0.017 |
| Week 2 PTSD | .0266 | 0.003 | 0.016 | 0.014 |
| Week 2 Somatic | -.0019 | 0.003 | -0.010 | -0.030 |
| Week 8 Pain | .0211 | 0.070** | 0.014 | 0.054* |
| Week 8 Depression | -.0010 | -0.006 | 0.016 | 0.016 |
| Week 8 PTSD | .0059 | 0.003 | 0.021 | 0.024 |
| Week 8 Somatic | -.0032 | 0.035 | 0.005 | 0.006 |
| ** Correlation is significant at the 0.01 level (2-tailed). | | | |  |
| * Correlation is significant at the 0.05 level (2-tailed). | | | |  |

**Supplementary Table 5.** Missing Data : Number (%) of participants have at least one flash survey for each of the 5 flash surveys.

| **N Total** | **Pain Flash Survey** | **Sleep Flash Survey** | **Avoidance Flash Survey** | **Anxiety Flash Survey** | **Somatic Flash Survey** |
| --- | --- | --- | --- | --- | --- |
| **2097** | 1903(91%) | 1839(88%) | 1847(88%) | 1807(86%) | 1871(89%) |

**Supplementary Table 6.** Loss to follow-up: Demographic characteristics and pre-trauma mental health outcomes between participants dropped/withdrew from the study and participants completed the 8-week survey.

|  | **Dropped and Withdrew** | **Completed** | **P-value** |
| --- | --- | --- | --- |
| **Age** |  |  |  |
| Mean (SD) | 32.7 (12.5) | 35.8 (13.1) | <0.001 |
| **Gender (Male)** | 321 (44.6%) | 791 (37.2%) | <0.001 |
| **Race** |  |  |  |
| Hispanic | 107 (14.9%) | 239 (11.2%) | 0.008 |
| Non-Hispanic White | 203 (28.2%) | 722 (34.0%) |  |
| Non-Hispanic Black | 376 (52.3%) | 1074 (50.5%) |  |
| Non-Hispanic Other | 31 (4.3%) | 81 (3.8%) |  |
| **Pre-trauma Moderate/Severe Pain** | 240 (33.4%) | 687 (32.3%) | 0.588 |
| **Pre-trauma Somatic Symptoms** |  |  |  |
| Mean (SD) | 4.14 (3.77) | 4.07 (3.57) | 0.684 |
| **Pre-trauma Depression** | 131 (18.2%) | 327 (15.4%) | 0.079 |
|  |  |  |  |

**Supplementary Table 7.** Parameter estimates of trajectory models for each latent construct. For Growth Trajectory, Linear means linear trajectory model, and Piecewise means linear piecewise trajectory model.

| **Latent Construct** | **Growth Trajectory** | **Intercept (I)** | **First Slope (S1)** | **Second Slope (S2)** | **Variance of Intercept** | **Variance of First Slope** | **Variance of Second Slope** | **Correlation of I and S1^2^** | **Correlation of I and S2^3^** |
| --- | --- | --- | --- | --- | --- | --- | --- | --- | --- |
| Pain | Piecewise | **6.12***** | **-0.650***** | **-0.100***** | **2.89***** | **0.380***** | **0.086***** | **0.049** | **-0.121*** |
| Depression | Piecewise | **1.37***** | **0.007** | **-0.026***** | **0.91***** | **0.027***** | **0.012***** | **-0.205**** | **-0.094** |
| Sleep Discontinuity | Piecewise | **1.90***** | **-0.053***** | **-0.026***** | **0.57***** | **0.021***** | **0.006**** | **-0.050** | **-0.087** |
| Nightmare | Piecewise | **1.16***** | **-0.046***** | **-0.013*** | **0.79***** | **0.013*** | **0.004*** | **-0.016** | **-0.142** |
| Avoidance | Piecewise | **1.82***** | **-0.033***** | **-0.051***** | **0.71***** | **0.026***** | **0.011***** | **-0.163** | **-0.307**** |
| Re-experience | Piecewise | **2.06***** | **-0.116***** | **-0.051***** | **0.88***** | **0.025***** | **0.012***** | **-0.170*** | **-0.195**** |
| Anxiety | Linear | **1.43***** | **-0.037***** | **NA** | **0.88***** | **0.006***** | **NA** | **-0.177***** | **NA** |
| Hyperarousal | Piecewise | **2.30***** | **-0.104***** | **-0.048***** | **0.52***** | **0.05** | **0.05** | **-0.056** | **0.139*** |
| Somatic Symptoms | Piecewise | **5.06***** | **-0.288***** | **-0.069***** | **3.88***** | **0.103***** | **0.012** | **0.118** | **-0.442** |
| Mental Fatigue^1^ | Piecewise | **5.34***** | **-0.234***** | **-0.087***** | **2.35***** | **0.156***** | **0.037***** | **0.164*** | **-0.151*** |

^1^Mental Fatigue are measured by three questions related to concentration, thinking and fatigue.

^2^Correlation between intercept and first slope

^3^Correlation between intercept and second slope

***** p-value<0.001; ** p-value<0.01; * p-value<0.05**

**Supplementary Table 8.** Number of latent classes identified for each latent construct

| **Latent Construct** | **Number of Latent Classes** |  | **Notes** |
| --- | --- | --- | --- |
| Pain | 4 |  | 4 latent classes have the best BIC. Variance and covariance of intercept and slope are constrained to be the same across latent classes. |
| Depression | 3 |  | 3 latent classes have the best BIC. Variance and covariance of intercept and slope are constrained to be the same across latent classes. |
| Sleep Discontinuity | 2 |  | 4 and 3 latent classes have smaller BIC, but factor covariance matrix is not positive definite |
| Nightmare | 4 |  | 4 latent classes have the best BIC. Variance and covariance of intercept and slope are constrained to be the same across latent classes. |
| Avoidance | 2 |  | 4 and 3 latent classes have smaller BIC, but one of the latent classes include less than 5% samples. |
| Re-experience | 4 |  | 4 latent classes have the best BIC. Variance and covariance of intercept and slope are constrained to be the same across latent classes. |
| Anxiety | 3 |  | 3 latent classes have the best BIC, but some parameter estimates are out of boundary. Variance and covariance of intercept and slope are constrained to be the same across latent classes. |
| Hyperarousal | 4 |  | 4 latent classes have the best BIC. Variance and covariance of intercept and slope are constrained to be the same across latent classes. |
| Somatic Symptoms | 4 |  | 4 latent classes have the best BIC. Variance and covariance of intercept and slope are constrained to be the same across latent classes. |
| Mental Fatigue^1^ | 3 |  | 4 latent classes have smaller BIC, but one of the latent classes include less than 5% samples. |

^1^Mental Fatigue are measured by three questions related to concentration, thinking and fatigue.

**Supplementary Figure 1. Correlation of intercepts**: Hit Head (left) vs Not Hit Head (right). During enrollment at the emergency department, participants were asked the question “Did you hit your head or experience a head injury during the event that brought you to the ER today?”.


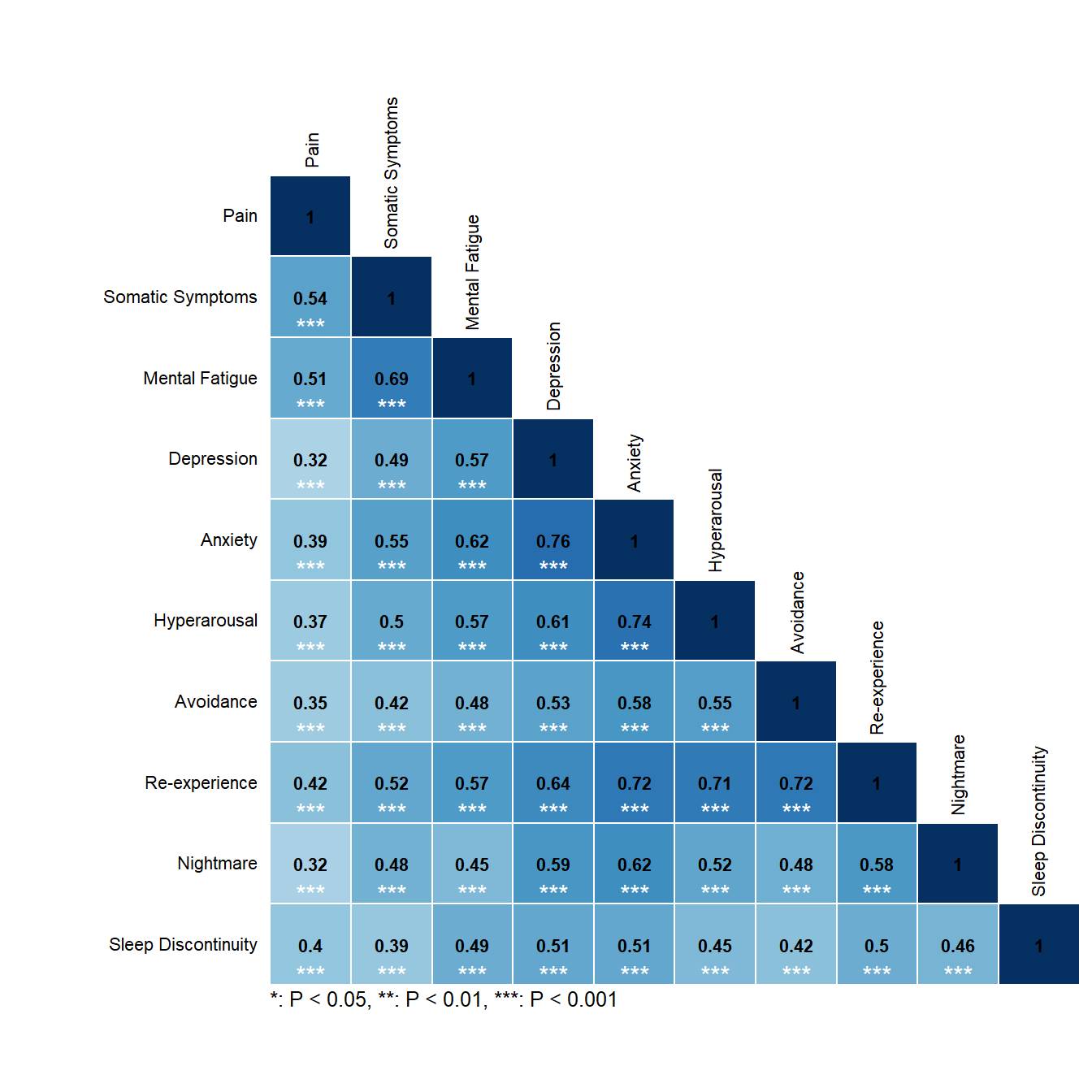

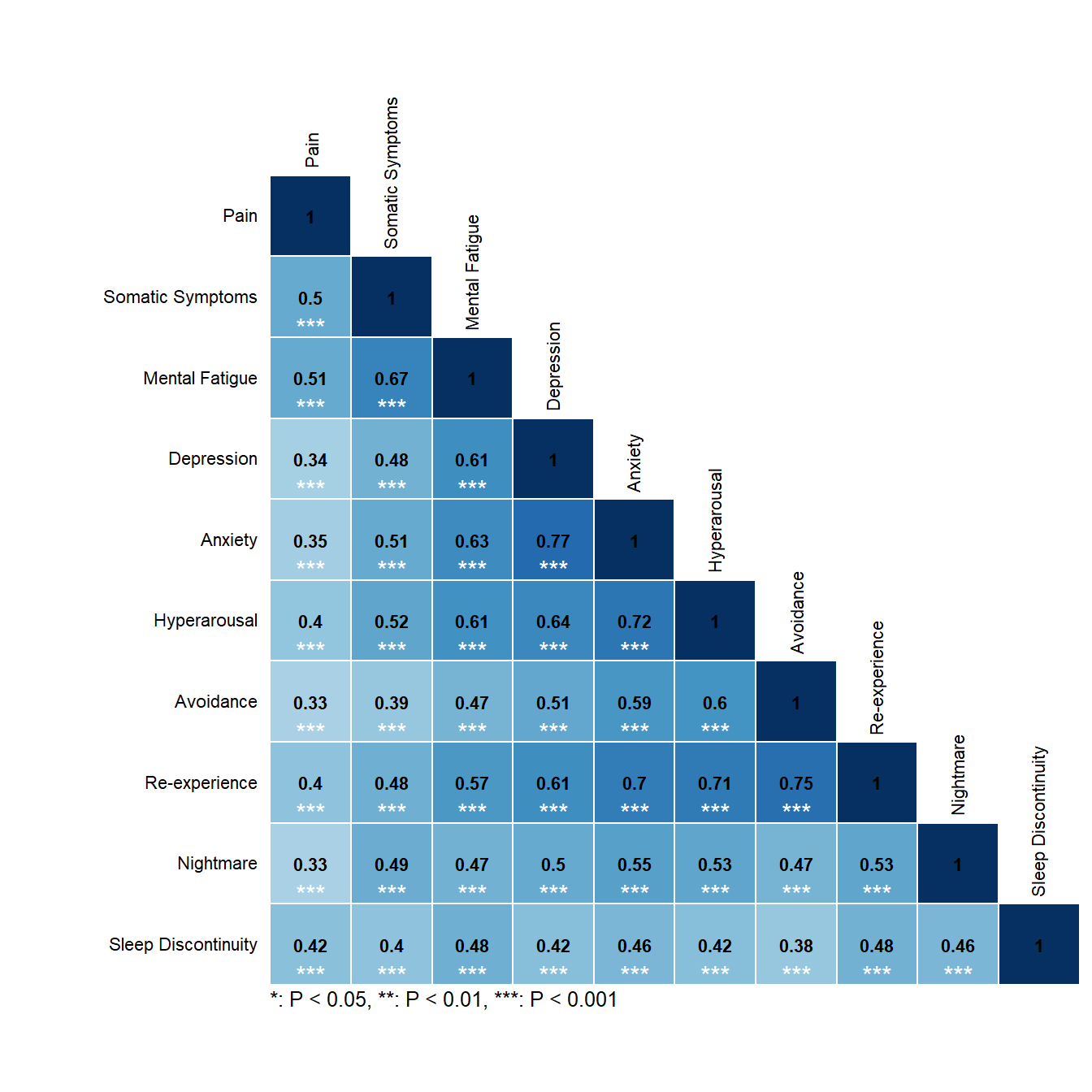


**Supplementary Figure 2**. Correlation of slopes: Hit Head (left) vs Not Hit Head (right). During enrollment at the emergency department, participants were asked the question “Did you hit your head or experience a head injury during the event that brought you to the ER today?”.


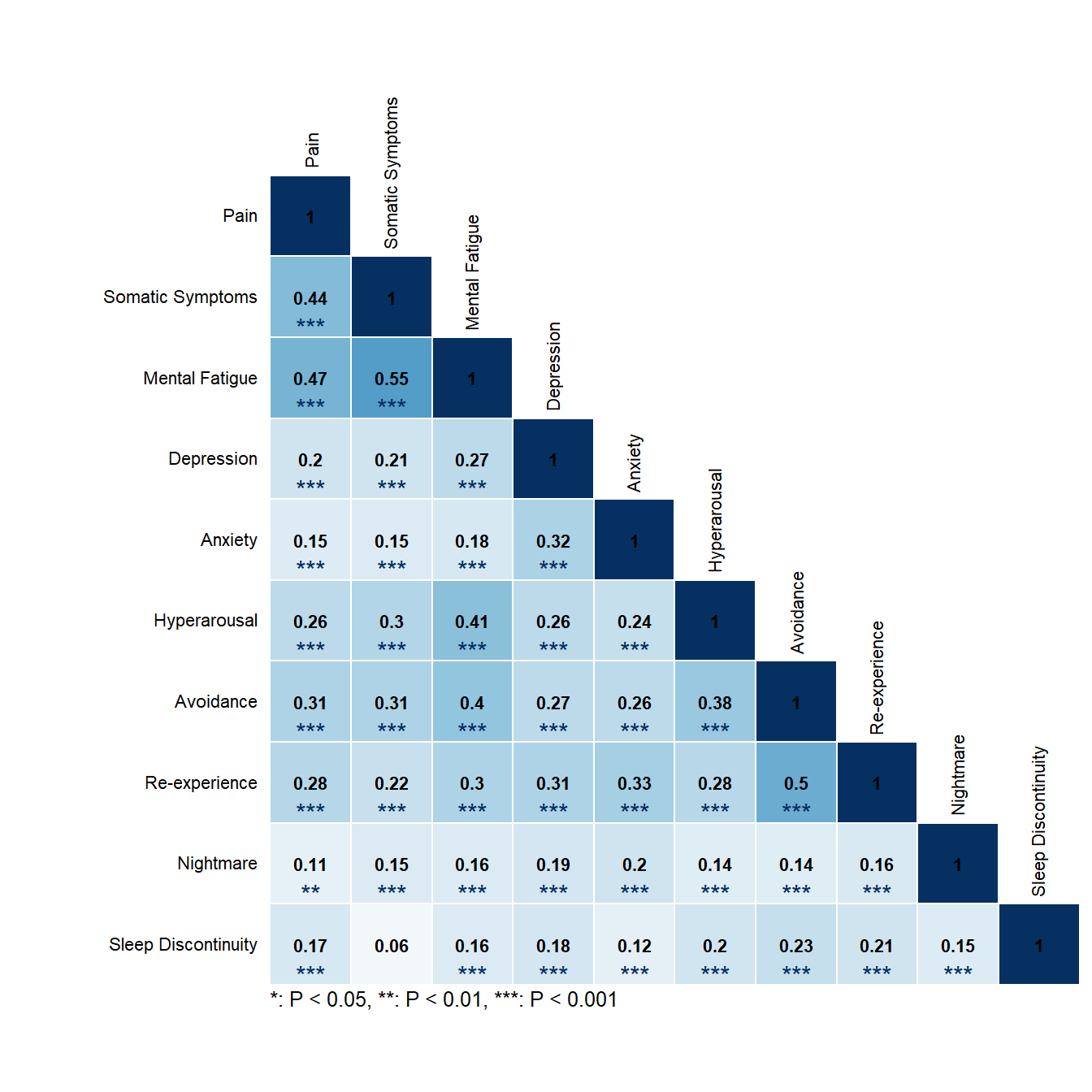

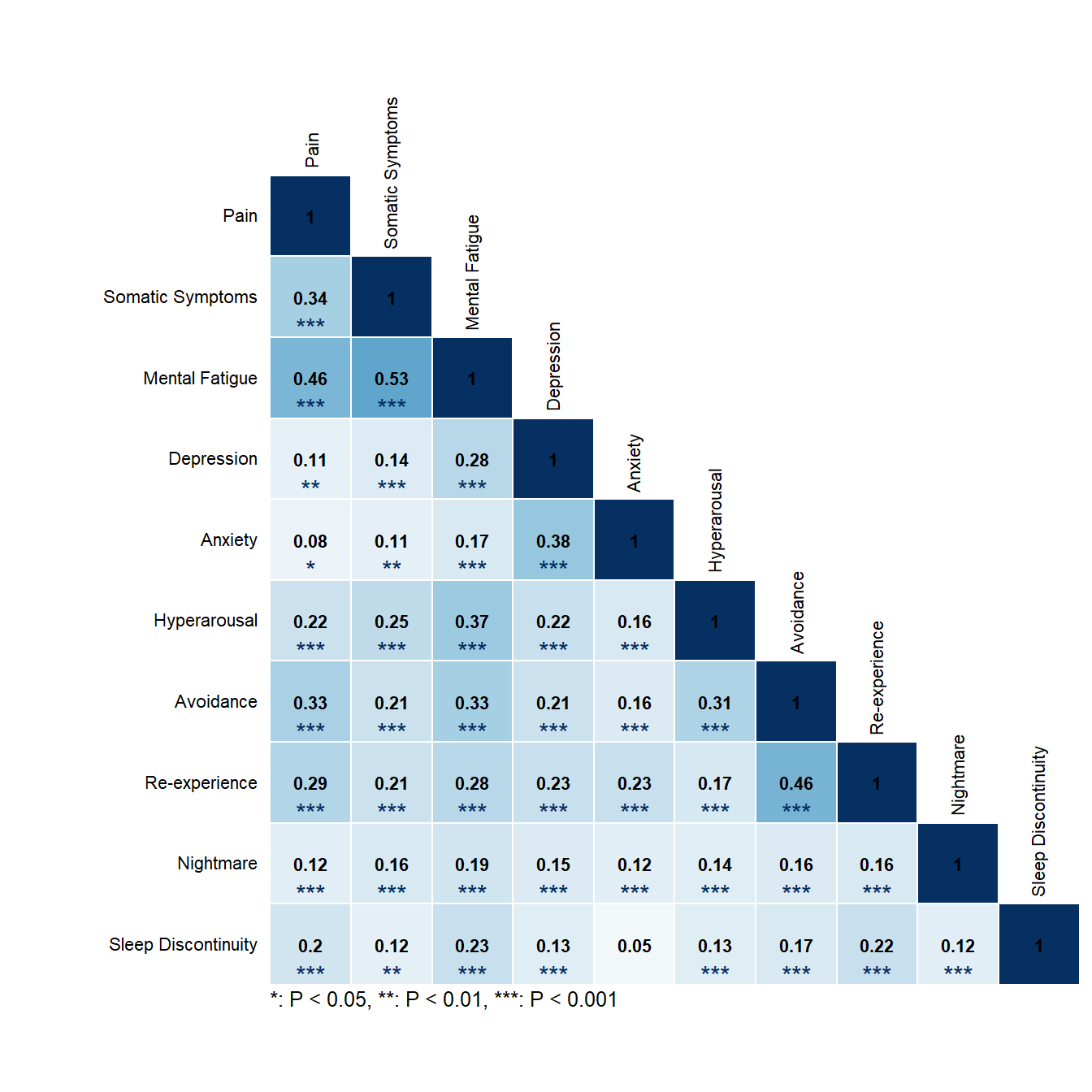


**Supplementary Figure 3. Screen capture of the online interactive visualization tool.**


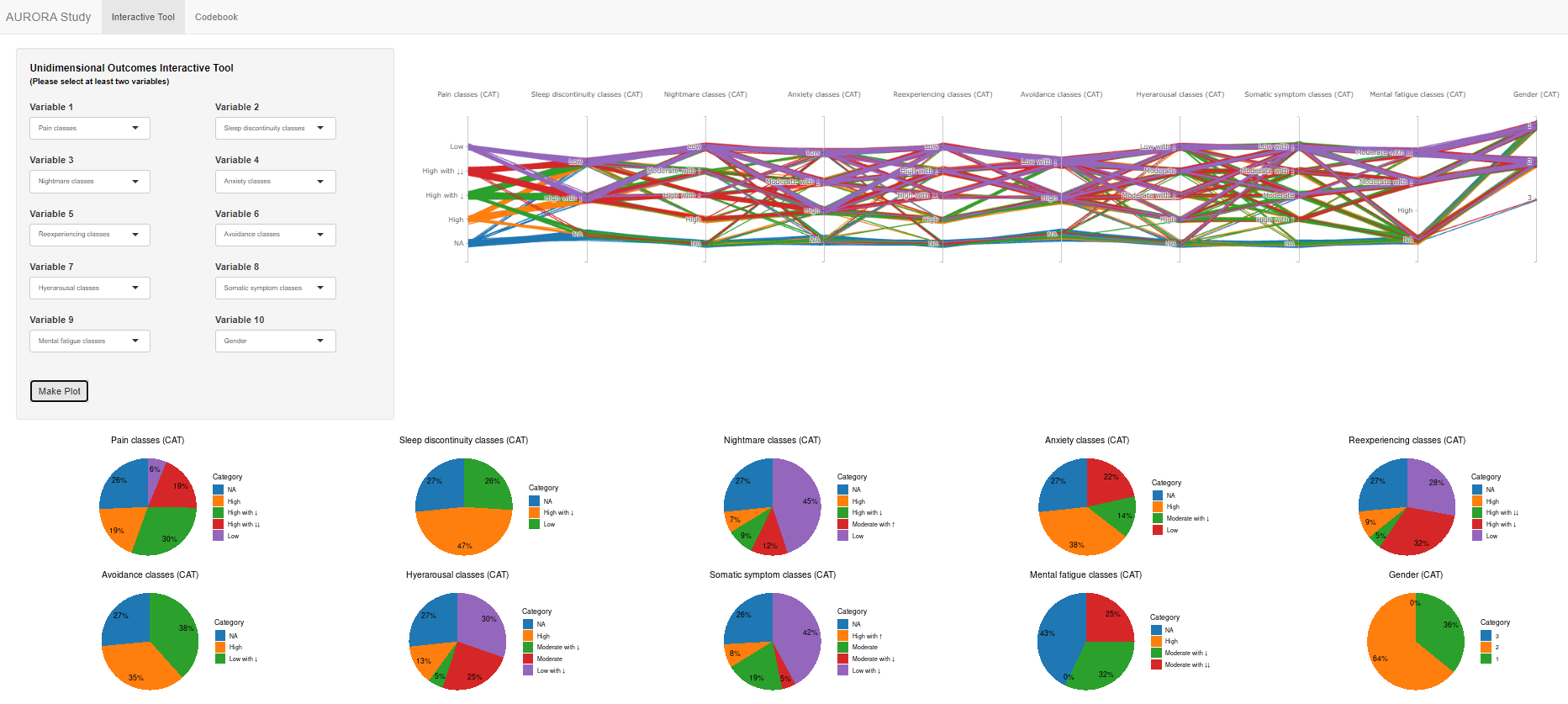


The online tool is an interactive visualization tool that was developed based on R Shiny (<http://itr.med.unc.edu/aurora/parcoord/>). The tool provides researchers with an interactive way to explore the relationship among the unidimensional RDoC outcomes and other related outcomes. Supplement Figure 6 displays a screen capture of the tool with sample output. The tool includes three parts: (1) a drop down menu that allow users to select the outcomes (Figure, top left); (2) a parallel coordination plot (Figure, top right); and (3) pie charts that displays proportions of different classes (Figure, bottom). The parallel coordination plot illustrates how different subgroups of participants, defined by the first outcome selected in the drop down menu, are distributed across other outcomes. In the default setting, the plot shows how the participants in the four pain subgroups (High, High with **↓**, high with**↓↓,** and Low) are distributed into other domain groups (e.g., sleep discontinuity, etc.). You can also select a further subset of participants on the plot by “brushing” along the vertical line for each outcome. For example, you can select the high pain and low sleep discontinuity group by making two brushes, one on each of the vertical lines for pain and sleep discontinuity. You can cancel the selection by left clicking on the vertical line. Whenever you select a subset of participants, the pie charts on the bottom will be updated automatically for the selected subset of participants. Data set used for this interactive tool includes demographic information, general health, traditional outcomes (e.g., Pain, PTSD, and Somatic) at ED, week 2 and week 8, childhood and life time trauma, substance use, and unidimensional outcomes. A code book is included along with the tool with a separate tab next to interactive tool (top left).
